# Transverse Myelitis in a Healthy Patient With Benign Paroxysmal Positional Vertigo: A Case Report Highlighting Inpatient Rehabilitation and Functional Gains

**DOI:** 10.7759/cureus.76490

**Published:** 2024-12-27

**Authors:** Alan J Faulks, Elora Mallick, Duc Chung, Tha Cha

**Affiliations:** 1 Physical Medicine and Rehabilitation, California Health Sciences University, Clovis, USA; 2 Palliative Care, California Health Sciences University, Clovis, USA; 3 Physical Medicine and Rehabilitation, San Joaquin Valley Rehabilitation Hospital, Fresno, USA

**Keywords:** 6-point scale, activity not attempted codes, benign paroxysmal positional vertigo, functional mobility performance, in-patient rehabilitation facility, intensive rehabilitation, self-care performance, transverse myelitis

## Abstract

Transverse myelitis (TM), a poorly understood neurological disorder, can manifest in various clinical scenarios. We report a unique case where TM presented in a background of benign paroxysmal positional vertigo (BPPV). The patient, an otherwise healthy female, experienced a rapid onset of symptoms, culminating in complete left-sided hemiparesis and exacerbation of BPPV characteristics. Following admission to an inpatient rehabilitation facility, significant enhancements in activities of daily living were observed, underscoring the pivotal role of intensive physical rehabilitation.

## Introduction

Transverse myelitis (TM) is defined as inflammation of the spinal cord, specifically within the myelin sheath [1]. TM often presents with rapid onset weakness, sensory deficits, and bowel/bladder dysfunction [2]. Any level of the spinal cord can be affected, but most commonly, the thoracic region is impaired. There are multiple causes of TM, but they can broadly be divided into these categories: idiopathic, postinfectious, systemic inflammation, or multifocal central nervous system disease. TM can be differentiated from conditions with overlapping symptoms, such as multiple sclerosis (MS) and cervical radiculopathy, through MRI findings and CSF analysis. MS typically has distinct CSF findings of oligoclonal bands not found in TM [3]. In contrast to cervical radiculopathy, TM typically demonstrates intramedullary spinal cord lesions on MRI, while cervical radiculopathy will reveal nerve root compression typically due to foraminal narrowing [4]. The standard of care and the first-line therapy for the treatment of TM is intravenous glucocorticoids. This case study describes a 51-year-old woman with a past medical history significant only for benign paroxysmal positional vertigo (BPPV) who presents with an unusual presentation of acute TM that is subsequently treated with medical management and four weeks of intensive, inpatient physical and occupational therapy rehabilitation. TM and BPPV are distinct clinical entities with no direct pathophysiological relationship. While both conditions involve the nervous system, BPPV affects the vestibular apparatus in the inner ear, whereas TM is an inflammatory condition affecting the spinal cord [5]. However, our patient demonstrates the onset of BPPV-related symptoms coinciding with neurologic and autonomic dysfunction.

## Case presentation

A 51-year-old Hispanic female with a past medical history significant for only BPPV presented to the emergency department with mid-thoracic pain radiating to bilateral upper and lower extremities and causing associated weakness. The patient denied any recent falls or inciting injury. She was discharged with a lidocaine patch after CT showed prominent posterior osteophytes at C6-C7 with mild left-sided foraminal narrowing.

The patient returned the same day with complete loss of sensation on the left side, numbness, tingling, BPPV-related symptoms, and loss of feeling of bladder fullness. The physical exam showed intact sensation to light touch on all extremities, hyperreflexia, and decreased strength in her left upper and lower extremities. She had normal strength in her right upper and lower extremities. Her white blood cell count was initially elevated at a value of 16.9. CRP was markedly elevated at 13.1, while ESR was within normal limits, suggesting an inflammatory cause of the patient’s symptoms rather than mechanical compression, as in cervical radiculopathy. Autoimmune and infectious workup was also within normal limits, including two negative blood cultures without growth, negative anti-neuromyelitis optica (anti-NMO) antibody (anti-aquaporin 4 ab), negative anti-myelin oligodendrocyte glycoprotein (anti-MOG) antibody, negative antinuclear antibody (ANA), normal methylmalonic acid levels, normal serum copper, negative Lyme titers, and West Nile serum negative. CSF studies showed scattered reactive mononuclear cells with no evidence of malignancy. CSF glucose was 79, and CSF protein was 50. Given the negative autoimmune and infectious workup, these findings suggest an idiopathic source of TM. Table 1 shows the lab values that were pertinent to her hospitalization.

**Table 1:** Pertinent lab values from hospitalization WBC: White blood cell count; ESR: Erythrocyte sedimentation rate; CRP: C-reactive protein; ANA: Antinuclear antibody; TSH: Thyroid stimulating hormone; NMO: Neuromyelitis optica; AQP4: Aquaporin 4; MOG: Myelin oligodendrocyte glycoprotein; PCR: Polymerase chain reaction; HIV: Human immunodeficiency virus; IgG: Immunoglobulin G; IgM: Immunoglobulin M; AB: Antibody; CSF: Cerebrospinal fluid; HSV: Herpes simplex virus; CMV: Cytomegalovirus; Enterovirus: Enterovirus; VZV: Varicella-zoster virus; IFA: Indirect immunofluorescence assay; CBA: Cell-based assay; GAD65: Glutamic acid decarboxylase 65; GFAP: Glial fibrillary acidic protein; mGluR1: Metabotropic glutamate receptor 1; NIF: Neuronal intermediate filaments; PCA: Purkinje cell antigen; AgNA: Anti-neuronal antigen

Name	Value	Reference Range
Serum		
WBC	16.9	4.0-11.0 10*3/uL
ESR	16	0-30 mm
CRP	13.1	<=3.0 mg/L
ANA Screen, IFA	Negative	Negative
Copper	112	70-175 mcg/dL
Thyroid Screen TSH	1.040	0.450-5.330 uIU/mL
Vitamin B12	553	211-911 pg/mL
Methylmalonic Acid	128	87-318 nmol/L
NMO/AQP4 FACS	Negative	Negative
MOG AB CBA	Negative	Negative
Infectious Disease - Serum		
SARS-CoV-2 by PCR	Not Detected	Not detected
*Mycoplasma pneumoniae* by PCR	Negative	Negative
Parainfluenza by PCR	Negative	Negative
Influenza A by PCR	Negative	Negative
Influenza B by PCR	Negative	Negative
RSV by PCR	Negative	Negative
*Human metapneumovirus* by PCR	Negative	Negative
Adenovirus by PCR	Negative	Negative
Rhinovirus by PCR	Negative	Negative
HIV AG AB Screen 4th Gen	Non-reactive	Non-reactive
Syphilis IgG Cascade	Nonreactive	Nonreactive
West Nile IgG	<1.30	<1.30 Antibody not detected
West Nile IgM	<0.90	<0.90 Antibody not detected
Lyme Ab Screen	<0.90	<0.90 Negative
Legionella Urinary Antigen	Negative	Negative
*Streptococcus pneumoniae* Urinary Antigen	Negative	Negative
Blood Cultures		
Blood Culture #1	Final: No growth	-
Blood Culture #2	Final: No growth	-
Cerebral Spinal Fluid		
CSF Glucose	79	35-70 mg/dL
CSF Protein	50	15-45 mg/dL
Culture, Cerebral Spinal Fluid	Final: no growth	-
Infectious Disease - CSF		
*Escherichia coli *K1 PCR	Not detected	Not detected
*Haemophilus influenzae* PCR	Not detected	Not detected
*Listeria monocytogenes* PCR	Not detected	Not detected
*Neisseria meningitidis* PCR	Not detected	Not detected
*S. agalactiae* PCR	Not detected	Not detected
*S. pneumoniae* PCR	Not detected	Not detected
*Cytomegalovirus* PCR	Not detected	Not detected
*Enterovirus* PCR	Not detected	Not detected
*Human herpesvirus* 6 PCR	Not detected	Not detected
HSV1 CSF PCR	Not detected	Not detected
HSV2 CSF PCR	Not detected	Not detected
*Human parechovirus *PCR	Not detected	Not detected
*Varicella-zoster virus* PCR	Not detected	Not detected
*Cryptococcus neoformans*/*gattii* PCR	Not detected	Not detected
Myelopathy, Autoimmune, Paraneoplastic - CSF		
Aquaporin 4 AB, CBA, CSF	Negative	Negative
MOG AB CBA, CSF	Negative	Negative
Amphiphysin Ab, CSF	Negative	Negative
AGNA-1, CSF	Negative	Negative
ANNA-1, CSF	Negative	Negative
ANNA-2, CSF	Negative	Negative
ANNA-3, CSF	Negative	Negative
AP3B2 IFA, CSF	Negative	Negative
CRMP-5-IgG Western Blot, CSF	Negative	Negative
DPPX Ab IFA, CSF	Negative	Negative
GABA-B-R Ab CBA, CSF	Negative	Negative
GAD65 Ab Assay, CSF	Negative	Negative
GFAP IFA, CSF	Negative	Negative
mGluR1 Ab IFA, CSF	Negative	Negative
Neurochondrin IFA, CSF	Negative	Negative
NIF IFA, CSF	Negative	Negative
PCA-1, CSF	Negative	Negative
PCA-2, CSF	Negative	Negative
Septin-7 IFA, CSF	Negative	Negative

**Figure 1:**
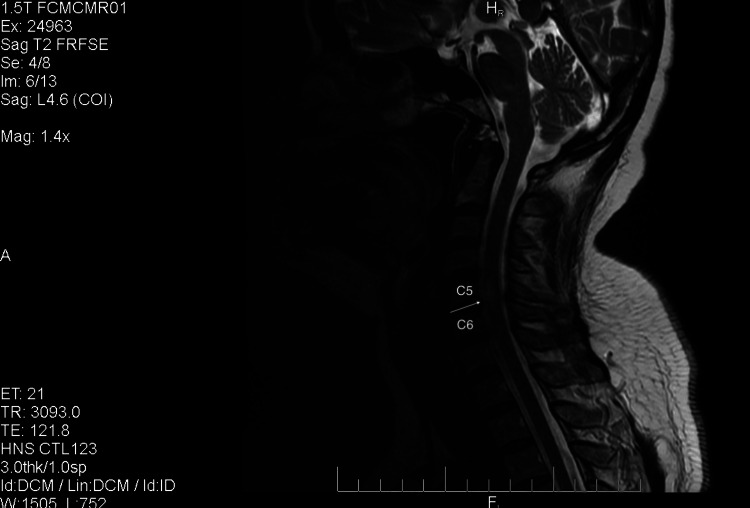
MRI of the patient’s cervical spine, showing T2-hyperintense signal over the cervical spine, consistent with edema at the C5-C6 level.

MRI without contrast revealed a T2-hyperintense signal over the cervical spine consistent with edema of C5-C6, as shown in Figure 1. The patient was diagnosed with TM and was given IV pulse therapy with high-dose methylprednisolone 1g daily for the first five days, followed by plasma exchange therapy for the subsequent five days.

Throughout the patient's 13-day length of stay, she was consistently hypotensive, with her systolic blood pressure ranging in the 70s-100s with unclear etiology. She was also unable to move her left lower extremity and clench her fingers on her left upper extremity.

The patient was then admitted for inpatient rehabilitation, consisting of at least three hours daily of combined physical therapy (PT) and occupational therapy (OT) for the next four weeks. Before admission, the patient was completely independent, highlighting the significant morbidity and abruptness of her clinical condition. During her initial evaluation, she was found to have no muscle movement or contractions in her left lower extremity and minimal movement and contractions in her left upper extremity. Throughout her rehabilitation, she continued to experience symptoms from her BPPV, such as dizziness and nausea during her therapy sessions. Tables 2-3 show the patient’s self-care performance during OT and PT evaluations at admission and discharge.

**Table 2:** Occupational Therapy Evaluation Self-Care Performance scores at admission and discharge 01: Dependent, 02: Substantial/maximal assistance, 03: Partial/moderate assistance, 04: Supervision or touching assistance, 05: Setup or clean-up assistance, 06: Independent, 88: Not attempted due to a medical condition or safety concerns. More information regarding these scores can be found in Table 4.

Occupational Therapy Evaluation Self-Care Performance		
	Admission	Discharge
Eating	04	05
Oral hygiene	04	05
Toileting hygiene	01	03
Shower/bathe self	02	03
Upper body dressing	02	05
Lower body dressing without footwear	01	03
Putting on/taking off footwear	01	03
Shower transfer	01	04

**Table 3:** Physical Therapy Evaluation Self-Care Performance scores at admission and discharge 01: Dependent, 02: Substantial/maximal assistance, 03: Partial/moderate assistance, 04: Supervision or touching assistance, 05: Setup or clean-up assistance, 06: Independent, 88: Not attempted due to a medical condition or safety concerns. More information regarding these scores can be found in Table 4.

Physical Therapy Evaluation Functional Mobility Performance		
	Admission	Discharge
Roll left and right	01	06
Sit to lying	01	04
Lying to sitting on side of bed	02	03
Sit to stand	02	04
Chair/bed to chair transfer	01	05
Toilet transfer	01	04
Walk 10 feet	88	01
Walk 10 feet on uneven surfaces	88	88
Walk 50 feet with two turns	88	88
Walk 150 feet once standing	88	88
1 step curb	88	88
4 steps (ability to go up and down with or without a rail)	88	88
12 steps (ability to go up and down with or without a rail)	88	88
Wheel 50 feet with two turns	01	04
Wheel 150 feet	88	88
Car transfer	88	04
Picking up object (bend-stoop from a standing position to pick up a small object, such as a spoon, from the floor)	88	88
Does patient use a wheelchair or scooter?	No	Yes

Comparisons of initial and final OT and PT evaluations of functional ability showed noteworthy improvements, with tasks like toilet hygiene increasing from 01 to 03, rolling left and right from 01 to 06, sitting to lying down from 01 to 04, and chair/bed to chair transfer from 01 to 05. However, activities like walking 10 feet on an uneven surface, 50 feet with two turns, and 150 feet once standing remained unchanged at 88. This scoring indicates they were not attempted due to safety concerns caused by her continued left upper and lower extremity weakness. The patient was discharged from inpatient rehabilitation with plans to continue OT and PT in an outpatient setting.

## Discussion

TM can affect men and women equally and can affect patients of all ages [6,7]. However, a bimodal peak is seen between ages 10 to 19 and 30 to 39 [7]. There are approximately one to eight new cases per million people annually. There is no difference in occurrence between European, American, African, and Asian-born populations [8].

The exact mechanisms underlying the initiation and progression of TM have yet to be fully understood and may vary among individuals. Specific triggers and immune responses involved in TM can differ, leading to heterogeneous clinical presentation and outcomes. Until an MRI of the spinal cord can be done, a differential diagnosis for TM may include demyelination conditions like MS and NMO, infections such as herpes zoster and herpes simplex virus, and other inflammatory disorders such as systemic lupus erythematosus and neurosarcoidosis [9]. This case report presents a unique case of a patient who only has a background of recurrent tension headaches and BPPV. Symptoms of BPPV include dizziness, loss of balance, nausea and vomiting, hearing loss, and vision problems [10]. BPPV is not known to be a risk factor for developing other conditions, and there is no known association between BPPV and TM. When assessing the patient's symptoms of vertigo with bilateral upper and lower extremity weakness, it is reasonable to consider other differentials, such as MS or cerebrovascular accidents, particularly in the brainstem or cerebellum [11,12]. However, the functional presentation of the patient and initial CT findings of cervical osteophytes suggested a more likely differential of cervical radiculopathy that could be conservatively treated with 5% topical lidocaine [13,14].

Due to the severity of neurological symptoms experienced by the patient, she was transferred to an inpatient rehabilitation facility (IRF). The goal of rehabilitation in this setting is to improve a patient's functional ability in performing activities of daily living by improving range of motion, creating compensatory strategies, and relieving pain [15]. Physical, occupational, and speech therapy are the primary services offered at an IRF, where patients can work on improving gross and fine motor skills and addressing any speech pathology issues. Physical and occupational rehabilitation goals are represented by domains of the 6-Point Scale and Activity Not Attempted Codes necessary to represent Medicare patients [16]. This scale, ranging from 01 (dependent) to 06 (independent), is used to establish a patient's baseline and measure relevant progress. Table 4 shows the 6-Point Scale and Activity Not Attempted Codes with specific definitions of each code.

**Table 4:** The 6-Point Scale and Activity Not Attempted Codes from Centers for Medicare and Medicaid Services

The 6-Point Scale and Activity Not Attempted Codes		
Coding: Safety and Quality of Performance - If helper assistance is required because patient’s/resident’s performance is unsafe or of poor quality, score according to amount of assistance provided.		
06	Independent	Patient/resident safely completes the activity by him/herself with no assistance from a helper.
05	Setup or clean-up assistance	Helper sets up or cleans up; patient/resident completes activity. Helper assists only prior to or following the activity.
04	Supervision or touching assistance	Helper provides verbal cues and/or touching/steadying and/or contact guard assistance as patient/resident completes activity. Assistance may be provided throughout the activity or intermittently.
03	Partial/moderate assistance	Helper does LESS THAN HALF the effort. Helper lifts, holds or supports trunk or limbs, but provides less than half the effort.
02	Substantial/maximal assistance	Helper does MORE THAN HALF the effort. Helper lifts or holds trunk or limbs and provides more than half the effort.
01	Dependent	Helper does ALL of the effort. Patient/resident does none of the effort to complete the activity. Or, the assistance of 2 or more helpers is required for the patient/resident to complete the activity.
If activity was not attempted, code reason:		
07	Patient/resident refused	
09	Not applicable	Not attempted and the patient/resident did not perform this activity prior to the current illness, exacerbation, or injury.
10	Not attempted due to environmental limitations	(e.g., lack of equipment, weather constraints)
88	Not attempted due to medical condition or safety concerns	

Before her hospital admission, the patient was completely independent in her daily activities. Coming to the IRF, she had deficits related to BPPV and new-onset left upper and lower extremity weakness. Therefore, she had significant regression in her function, and her initial rehabilitation started at a baseline of being almost entirely dependent. High-intensity rehabilitation of three hours a day for the next four weeks resulted in significant improvement in several PT and OT domains due to increased upper and lower extremity muscle strength and movement. Notably, the patient improved to at least a 04 in most domains, including function of the upper extremities, meaning she could perform tasks with only intermittent assistance, such as verbal or physical cues. Activities done during PT and OT included bed mobility, squat pivot transfers, static and dynamic standing balance, muscle strengthening and stretching exercises, limited gait training, neuromuscular re-education, fine motor control activities, energy conservation techniques, and caregiver training.

Other rehabilitation domains, particularly those related to lower extremity movement, remained at an 88, as testing the patient’s mobility with walking and stairs was unsafe due to her hemiparesis. However, during therapy sessions, the patient was noted to gain some left lower extremity movement against gravity, which is a stark improvement from the lack of any muscle contractions during her initial evaluation. Through gait therapy, she was eventually able to walk up to 40 feet with the assistance of a front wheel walker and varying levels of assistance for facilitation of her left leg and advancement. The patient also learned how to use a wheelchair during her rehabilitation course, representing an improvement from her baseline at admission to the IRF. Unfortunately, due to her left arm weakness, her overall mobility with the wheelchair was also limited.

After completing this intensive rehabilitation program, the patient transitioned to an outpatient rehabilitation program. This demonstrates that she achieved a level of functionality that eliminates the need for more dependent Home Health PT or OT services or placement in a skilled nursing facility [17,18]. It is important to recognize the significance of making adequate progress during admission at an IRF, as this directly correlates with caregiver burden [19]. This concept of caregiver burden is defined as the level of multifaceted strain the caregiver perceives from caring for a family member or loved one over time [20]. Therefore, the closer a patient comes to achieving independence in their intensive rehabilitation program, the greater the reduction in caregiver burden will be [21]. By achieving a level of independence that requires only intermittent assistance (04), this patient’s caregiver will experience significantly less burden compared to having to assist with every step of every activity (01). Even on the rehabilitation scales relevant to walking, using a wheelchair independently will reduce the caregiver's burden in daily transportation. Overall, the quality and success of inpatient rehabilitation are crucial for reducing caregiver burden and enhancing overall well-being, particularly in patients with spinal cord injuries [22].

## Conclusions

In conclusion, we present a case of rapid-onset TM that underscores its debilitating nature and the importance of adjuvant therapy with pharmacologic interventions and intensive physical rehabilitation. While the etiology of the disease often remains unknown, optimal functional outcomes can be achieved through intensive physical rehabilitation. Our patient experienced significant improvements with four weeks of daily three-hour OT and PT sessions at an IRF. Specifically, gains were observed in proximal mobility and wheelchair utilization, highlighting the efficacy of the intervention. However, many tasks related to lower extremity mobility remained untestable or dependent on assistance due to the safety concerns from the patient’s unresolved hemiparesis. By establishing a concrete baseline for activities of daily living, our approach not only promotes patient independence but also alleviates the burden on caregivers, emphasizing the importance of prompt and comprehensive rehabilitative care. Continued monitoring of rehabilitation progress is essential to assess the long-term functional outcomes achievable through physical rehabilitation in future cases of TM.
